# The non-linear link between non-high-density lipoprotein to high-density lipoprotein ratio and the risk of stroke in middle-aged and older adults in Chinese: a prospective cohort study from the China health and retirement longitudinal study

**DOI:** 10.3389/fendo.2023.1303336

**Published:** 2024-01-15

**Authors:** Lanbo Wang, Yong Han, Changchun Cao, Haofei Hu, Han Li

**Affiliations:** ^1^ Department of Radiology, Shengjing Hospital of China Medical University, Shenyang, Liaoning, China; ^2^ Department of Emergency, Shenzhen Second People’s Hospital, Shenzhen, Guangdong, China; ^3^ Department of Emergency, The First Affiliated Hospital of Shenzhen University, Shenzhen, Guangdong, China; ^4^ Department of Rehabilitation, Shenzhen Dapeng New District Nan’ao People’s Hospital, Shenzhen, Guangdong, China; ^5^ Department of Nephrology, Shenzhen Second People’s Hospital, Shenzhen, Guangdong, China; ^6^ Department of Nephrology, The First Affiliated Hospital of Shenzhen University, Shenzhen, Guangdong, China; ^7^ Department of Neurosurgery, Shengjing Hospital of China Medical University, Shenyang, Liaoning, China

**Keywords:** stroke, non-high-density lipoprotein to high-density lipoprotein ratio, non-linear association, Cox proportional-hazards regression, sensitivity analysis

## Abstract

**Objective:**

This study aims to assess the association between the non-HDL-c/HDL-c ratio and stroke risk among middle-aged and older adults participating in the China Health and Retirement Longitudinal Study (CHARLS).

**Methods:**

This study conducted a prospective cohort analysis, enrolling a total of 10,183 participants who met the designated criteria from CHARLS between 2011 and 2012. We then used the Cox proportional-hazards regression model to explore the relationship between baseline non-HDL-c/HDL-c ratio and stroke risk. Using a Cox proportional hazards regression with cubic spline function, we were able to identify the non-linear relationship between the non-HDL-c/HDL-c ratio and stroke occurrence. A series of sensitivity analyses were also carried out.

**Results:**

The average age of the participants included in this study was 59.16 ± 9.35 years, and 4,735 individuals (46.68%) were male. Over a median follow-up period of 7.0 years, a total of 1,191 people (11.70%) experienced a stroke. Using a Cox proportional hazards regression model that was fully adjusted, we found no statistically significant correlation between the non-HDL-c/HDL-c ratio and the risk of stroke (HR=1.022; 95% CI 0.964, 1.083). Nevertheless, we did observe a non-linear relationship and saturation effect between the non-HDL-c/HDL-c ratio and stroke. Employing a two-piece Cox proportional hazards regression model and a recursive algorithm, we determined an inflection point of 2.685 for the non-HDL-c/HDL-c ratio. In instances where the non-HDL-c/HDL-c ratio fell below 2.685, for every 1-unit decrease in the non-HDL-c/HDL-c ratio, the likelihood of stroke decreased by 21.4% (HR=1.214, 95% CI: 1.039-1.418). In contrast, when the non-HDL-c/HDL-c ratio exceeded 2.685, there was no statistically significant change in the risk of stroke for each unit decrease in the non-HDL-c/HDL-c ratio (HR: 0.967, 95% CI: 0.897-1.042). The consistency of these findings across multiple sensitivity analyses suggests their robustness.

**Conclusion:**

This study unveils a non-linear relationship between the non-HDL-c/HDL-c ratio and stroke risk in middle-aged and older adults in China. Specifically, when the non-HDL-c/HDL-c ratio was below 2.685, a significant and clearly positive association with stroke risk was observed. Additionally, maintaining the non-HDL-c/HDL-c ratio below 2.685 could potentially lead to a substantial reduction in the risk of stroke.

## Introduction

Stroke is a significant global health concern, one of the leading causes of death and disability (1). In China, the prevalence of stroke among adults aged 40 years and above stands at 2.06%, with an annual increase of 8.3% (2). Recent findings from a national survey in China revealed that the long-term disability rate five years after stroke was 45% (3). Considering the growing population of older adults in China, it becomes imperative to identify risk factors for stroke in order to alleviate the burden on society. Previous studies have demonstrated a correlation between prevalent chronic ailments, including diabetes mellitus, hypertension, heart diseases, dyslipidemia, and chronic kidney disease, and a heightened susceptibility to stroke among middle-aged and elderly individuals (4, 5). Nevertheless, it is important to note that conventional risk factors alone are insufficient in comprehensively elucidating all the potential risks associated with stroke (6–8). Consequently, there exists a pressing clinical imperative to investigate further modifiable risk factors that may contribute to the occurrence of stroke.

Non-high-density lipoprotein cholesterol (non-HDL-c) concentration is a composite marker of several atherogenic lipoproteins, including low-density lipoprotein (LDL), very-low-density lipoprotein (VLDL), intermediate-density lipoprotein (IDL), and lipoprotein (a). That is to say, non-HDL-c encompasses all cholesterol present in lipoproteins other than high-density lipoprotein cholesterol (HDL-c) (9). Extensive research has already established non-HDL-c as a robust and independent predictor of cardiovascular disease (CVD) risk, warranting its consideration as a secondary target for lipid-lowering therapy in individuals with atherosclerotic cardiovascular disease or those at high risk (10–12). More recently, attention has been drawn to the non-HDL-c to HDL-c ratio (non-HDL-c/HDL-c ratio), which exhibits a significant correlation with metabolic syndrome (13). The non-HDL-c/HDL-c ratio, which serves as a recently developed composite indicator of atherogenic lipids, encompasses information pertaining to both atherogenic and anti-atherogenic lipid particles (14). Numerous studies published in the literature have provided evidence that the non-HDL-c/HDL-c ratio surpasses conventional lipid parameters in evaluating intracranial atherosclerosis, coronary atherosclerosis, and arterial stiffness. Therefore, the non-HDL-c/HDL-c ratio emerges as a valuable lipid parameter for assessing cardiovascular and cerebrovascular disease risk (14–16). However, limited research exists regarding the association between the non-HDL-C/HDL-C ratio and stroke risk.

A recent study in China observed a positive association between the non-HDL-c/HDL-c ratio and stroke risk (HR: 1.24, 95% CI: 1.01-1.52, P=0.036) (17). However, it is important to note that this study only adjusted for age, smoking, gender, hypertension, body mass index (BMI), drinking, diabetes, and high-sensitivity C-reactive protein (hs-CRP) when analyzing the relationship between the non-HDL-c/HDL-c ratio and stroke. Other significant factors known to influence stroke, such as renal function (18), triglyceride (TG) levels (19), uric acid (UA) levels (20), platelet count (PLT) (21), and hemoglobin concentration (HGB) (22), were not accounted for in the analysis. Furthermore, the nature of the relationship between the non-HDL-c/HDL-c ratio and stroke, whether it is linear or non-linear, requires further investigation.

In this study, our objective is to elucidate the association between the non-HDL-c/HDL-c ratio and stroke risk by analyzing data from the China Health and Retirement Longitudinal Study (CHARLS), a comprehensive nationwide survey that provides representative information.

## Methods

### Study design

This cohort study harnessed data from the CHARLS, with its baseline survey carried out from 2011 to 2012. The study’s follow-up period extended until the cut-off point in 2018. CHARLS is a nationally representative cohort study that is still running (23). The independent variable in this study was the non-HDL-c/HDL-c ratio, while the outcome variable consisted of incident stroke categorized as either stroke or non-stroke.

### Data source and study population

The data for this study were obtained from the CHARLS, an ongoing nationwide population-based research project aimed at assessing economic, social, and health statuses (23). During the baseline survey conducted between 2011 and 2012, 17,708 participants from 450 communities in 150 districts and 28 provinces across China were enrolled in CHARLS through face-to-face household interviews. The selection of participants involved the utilization of a multistage stratified probability-proportional-to-size sampling method (23, 24). Subsequent follow-up interviews were conducted with participants every two years (24). The CHARLS investigation was authorized by the Peking University Biomedical Ethics Review Board (IRB00001052-11015), and all participants provided written informed consent (24). For access to the data and relevant information of this study, it is available for download on the CHARLS project website (http://charls.pku.edu.cn/).

**Figure 1:**
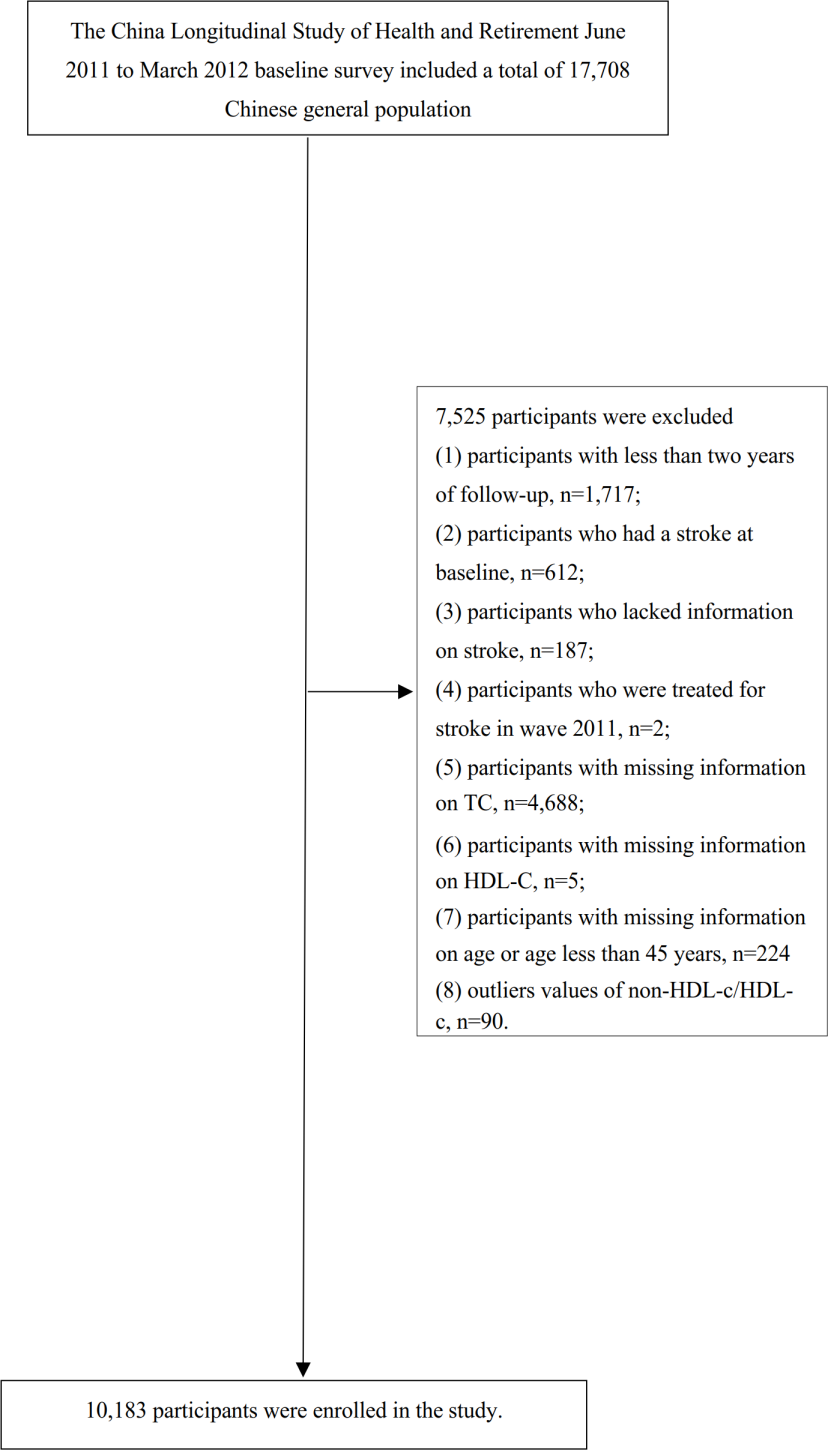
Flowchart of study participants. Illustrates the participant inclusion process. A total of 17,708 individuals were initially included in the baseline survey conducted from 2011 to 2012. Following the application of exclusion criteria, the final analysis consisted of 10,183 subjects included in this study.

Our study utilized data from four waves of CHARLS (2011, 2013, 2015, and 2018). The baseline survey conducted in 2011-2012 included a total of 17,708 participants. To ensure the reliability of our analysis, we followed a specific exclusion process. Firstly, participants with less than two years of follow-up (n=1,717) were excluded. Secondly, individuals who had a stroke at baseline (n=612), lacked information on stroke (n=187), or received stroke treatment in wave 2011 (n=2) were also excluded. Thirdly, participants with missing total cholesterol (TC) data (n=4,688) and missing information on HDL-c (n=5) were excluded from the analysis. Additionally, participants with missing information on age or with age less than 45 years (n=224) were excluded as well. Moreover, individuals with extreme values of the non-HDL-c/HDL-c ratio (outside the range of means plus or minus three standard deviations) (n=90) were excluded (25). Ultimately, a total of 10,183 participants were included in the final analysis. Please refer to **Figure 1** for a visual representation of the participant selection procedure.

### Variables

#### non-HDL-c/HDL-c ratio

The non-HDL-c/HDL-c ratio was recorded as a continuous variable. The calculation procedure for the non-HDL-c/HDL-c ratio is detailed below. The non-HDL-c/HDL-c ratio is derived by dividing the non-HDL-c value (measured in mg/dL) by the HDL-c value (measured in mg/dL). By deducting the HDL-c value from the total cholesterol (TC) measurement, the non-HDL-c value is derived (26). It is important to note that information regarding TC and HDL-c was collected during the baseline survey conducted in 2011-2012.

#### Diagnosis of stroke

Participants who did not report a stroke episode during the baseline survey but mentioned experiencing a stroke during the follow-up period were considered to have had a stroke event. As previously mentioned (23), standardized questions were used to collect information about stroke events, including: (i) Did your doctor inform you that you had been diagnosed with a stroke? (ii) When were you first diagnosed or became aware of the disease yourself? (iii) Are you currently receiving any follow-up treatment for your stroke? If an individual responded positively during the follow-up, they were classified as having received a first stroke diagnosis, and the self-reported time was recorded as the stroke onset. The time of the event was determined by subtracting the baseline survey time from the stroke onset time. In cases where the participant did not experience a stroke during any of the follow-up visits, the event onset time was calculated as the time of the last survey minus the baseline survey time.

#### Covariates

Covariates were selected based on previous research and our clinical knowledge (17, 23, 27). The following factors were included as covariates: (i) categorical variables: diabetes mellitus (DM), sex, hypertension, chronic lung disease (CLD), smoking status, coronary heart disease (CHD), drinking status, chronic kidney disease(CKD), daily activity, antihypertensive drug use, antihyperglycemic drug use, antihyperlipidemic drug use, malignant tumors, mental disease,; (ii) continuous variables: age, white blood cell (WBC), hs-CRP, HGB, blood urea nitrogen (BUN), PLT, body mass index (BMI), diastolic blood pressure (DBP), TG, systolic blood pressure (SBP), UA, hemoglobin A1c (HbA1c), and serum creatinine (Scr).

### Data collection

#### Questionnaire

Research center sent two interviewers to each county-level unit to interview about 72 households located in three communities. The checklist can refer to the study guide published by the research team (23). During the baseline and follow-up surveys, trained staff administered a standardized questionnaire to collect information about medical histories, sociodemographic characteristics, and lifestyle factors. These factors included sex (1 = male, 2 = female), age, physician-diagnosed chronic diseases (such as hypertension, diabetes mellitus, CLD, CHD, CKD, malignant tumors, mental disease), medication use (such as antihypertensive, hypoglycemic, and lipid-lowering drugs), as well as lifestyle and health-related behaviors (smoking, daily activity, alcohol consumption). Interviewers received training from CHARLS staff at Peking University to conduct interviews in respondents’ homes using computer-assisted personal interview techniques (23).

The term “chronic disease” was operationally defined as either a self-reported medical history of the disease or the current receipt of treatment for the disease (23). Smoking status was categorized into three distinct groups according to individuals’ smoking behavior: current smokers, individuals who have smoked in the past, and individuals who have never smoked. Similarly, drinking status was classified into three categories based on individuals’ drinking behavior: current drinkers, individuals who have previously consumed alcohol, and individuals who have never consumed alcohol (28). Daily activity was defined as engaging in at least 1.25 hours of vigorous activity per week, 2.5 hours of moderate-intensity activity per week, or a combination of both (equivalent to at least 600 metabolic equivalent minutes per week) (29). Medication use was defined as the use of antihypertensive medication, hypoglycemic medication, or lipid-lowering medication.

#### Physical examination and anthropometric measurements

The interviewers who conducted the county-level interviews described above also carried equipment for and conducted measurements of health functioning and performance in respondents’ households. The checklist can refer to the study guide published by the research team (23).

During the initial assessment, all participants underwent comprehensive physical examinations and anthropometric measurements administered by proficient examiners in accordance with established protocols. Standing height and body weight were recorded with participants attired in lightweight clothing and without footwear. BMI was computed by dividing weight in kilograms by the square of height in meters (kg/m^2). Blood pressure, encompassing both systolic and diastolic pressure, was assessed thrice at 45-second intervals in the left upper arm utilizing an automated electronic device (Omron™ HEM-7112, Omron Company, Dalian, China). The average values of the three measurements were employed for analysis (23).

#### Clinical and biochemical measurements

Prior to the commencement of the study, the participants were given instructions to abstain from consuming any food or beverages overnight. A 4-mL sample of venous blood was obtained, with the plasma and buffy coat being separated, while an additional 2-mL sample was specifically collected for the purpose of HbA1c analysis. The complete blood count (CBC) testing was conducted within a time frame of 1-2 hours after the collection of the samples. All blood samples were stored at a local laboratory at a temperature of 4°C and subsequently transported to the China Center of Disease Control (CDC) in Beijing within a period of two weeks, ensuring that a temperature of -80°C was maintained throughout the transportation process.

Measurements were taken from the frozen plasma or whole blood samples, including fasting plasma glucose (FPG) level, HbA1c, lipid panel (TC, HDL-c, LDL-c, and TG), Scr, hs-CRP, BUN, and UA. HbA1c levels were quantified employing Boronate affinity high-performance liquid chromatography (HPLC). TC, HDL-c, FPG, LDL-c, and TG concentrations were determined utilizing enzymatic colorimetric assays. Hs-CRP level was evaluated through an immunoturbidimetric assay. Scr levels were measured employing the rate-blanked and compensated Jaffe creatine method. UA levels were determined using the UA Plus method. Finally, BUN levels were assessed utilizing the Enzymatic UV method with urease (23, 30).

### Missing data handling

In our study, there were 228 (2.24%), 225 (2.21%), 1(0.01%), 12 (0.12%), 12 (0.12%), 1(0.01%), 2 (0.02%), 79 (0.78%), 226 (2.22%), 1451 (14.25%), 1451 (14.25%), 1491 (14.64%), 53 (0.52%), 89 (0.87%), 44 (0.43%), 32 (0.31%), 38 (0.37%), 50(0.49%), 35 (0.34%), 5945 (58.38%), 166 (1.63%), 11 (0.11%) participants with missing data for WBC, PLT, BUN, FPG, Scr, TG, LDL-c, HbA1c, HGB, SBP, DBP, BMI, hypertension, DM, malignant tumors, CLD, CHD, CKD, mental disease, daily activity, smoking status, drinking status, respectively. To address the issue of missing variables and ensure an accurate depiction of the statistical efficacy of the target sample during the modeling phase, multiple imputations were utilized to handle missing data in this study (31, 32). The imputation model incorporated a wide range of variables, including age, gender, drinking status, smoking status, WBC, PLT, CLD, hypertension, SBP, DBP, mental disease, malignant tumors, CHD, CKD, DM, Scr, daily activity, HbA1c, BUN, hs-CRP, UA, FPG, TG, LDL-c, BMI, and HGB. The analysis of missing data followed the assumption of missing-at-random (MAR) to ensure the validity of the imputation process (32).

### Statistical analysis

The participants were categorized into quartiles of the non-HDL-c/HDL-c ratio for stratification purposes. Baseline characteristics of continuous variables are presented as mean ± standard deviation (SD) for normally distributed variables, or as median (range) for variables with a skewed distribution. Categorical variables are expressed as percentages. To examine potential differences among the different non-HDL-c/HDL-c ratio groups, we employed the Kruskal-Wallis H test for variables with a skewed distribution, the One-Way ANOVA test for normally distributed variables, or the χ2 test for categorical variables.

To assess the existence of covariate colinearity, the variance inflation factor (VIF) was computed (33). The VIF formula is expressed as VIF = 1/(1-R^2^), where R^2^ denotes the R-squared value derived from a linear regression equation. In this equation, the dependent variable was the particular variable being examined, while the independent variables encompassed all other variables. If the VIF surpassed 5, it signified the presence of collinearity among the variables, leading to their exclusion from the multiple regression model (see **Supplementary Table S1**).

Following the collinearity screening, we proceeded to analyze three distinct models to evaluate the association between the non-HDL-c/HDL-c ratio and the risk of stroke. This assessment was conducted using both univariate and multivariate Cox proportional-hazards regression techniques. Model I represents the nonadjusted model, wherein no adjustments for covariates were made. Model II, on the other hand, is the minimally-adjusted model, where adjustments were solely made for sociodemographic variables such as age, gender, BMI, SBP, DBP, smoking and drinking habits, daily activity, hypertension, diabetes, CKD, CHD, mental illness, CLD, and malignant tumors. Model III was the fully-adjusted model, incorporating covariates presented in **Table 1**. These covariates included age, CKD, gender, diabetes, CHD, hypertension, mental disease, BMI, CLD, malignant tumors, daily activity, antihypertensive drug use, antihyperglycemic drug use, antihyperlipidemic drug use, SBP, DBP, smoking and drinking status, CRP, HGB, TG, Scr, HBA1c, BUN, UA, WBC, and PLT. Hazard ratios (HR) and corresponding 95% confidence intervals (CIs) were recorded. Adjustments for confounding variables were based on clinical knowledge and published reports (17, 23, 34), as well as the results of the collinearity screening, which revealed no collinearities among the variables (**Supplementary Table S1**). In order to verify the results obtained by treating the non-HDL-c/HDL-c ratio as a continuous variable and explore potential non-linear associations, we categorized it based on quartiles and calculated the P for trend. We also estimated the E-values of potential unobserved confounders between stroke risk and the non-HDL-c/HDL-c ratio (35). The E-value is an alternative approach to sensitivity analyses for unmeasured confounding in observational studies that avoids making assumptions that, in turn, require subjective assignment of inputs for some formulas (36). Specifically, an E-value analysis asks how strong the unmeasured confounding would have to be to negate the observed results (37). The E-value provides a quantitative measure to address this question by determining the minimum magnitude of association, in terms of the risk ratio, that an unmeasured confounder must have with both the treatment and outcome. It takes into account the influence of measured covariates, allowing for the evaluation of whether the observed treatment outcome association can be overridden. The E-value plays a crucial role in evaluating the robustness of study findings and assessing the plausibility of unmeasured confounding. If the strength of unmeasured confounding falls below the E-value threshold, it cannot overturn the main study result, preventing a shift towards a “no association” scenario (where the estimated risk ratio equals 1.0). By considering the magnitude of potential unmeasured confounding, E-values offer insight into the reliability of the main study outcome. The E-value provides a measure related to the evidence for causality, hence the name “E-value” (35). The formulas for the E-value for different effect measures, including continuous outcomes, are available (36), and the E-value has been implemented in freely available software and an online calculator (https://evalue.hmdc.harvard.edu/app/) (38).

**Table 1:** The baseline characteristics of participants.

non-HDL-c/HDL-c ratio	Q1(<2.07)	Q2(2.07-2.81)	Q3(2.81-3.75)	Q4(≥3.75)	P-value
**Participants**	2546	2545	2546	2546	
**Age (years)**	59.66 ± 9.72	59.29 ± 9.67	58.72 ± 8.90	58.97 ± 9.05	0.002
**WBC (10^9/L)**	5.92 ± 1.88	6.16 ± 2.80	6.31 ± 1.85	6.55 ± 1.89	<0.001
**PLT (10^9/L)**	200.00 ± 72.11	210.00 ± 74.04	213.68 ± 71.55	220.81 ± 72.03	<0.001
**BUN (mg/dL)**	16.27 ± 5.19	15.82 ± 4.59	15.45 ± 4.30	15.50 ± 4.32	<0.001
**FPG (mg/dL)**	103.03 ± 26.01	105.33 ± 26.15	109.53 ± 36.04	120.11 ± 45.28	<0.001
**Scr (mg/dL)**	0.77 ± 0.34	0.77 ± 0.19	0.78 ± 0.18	0.81 ± 0.20	<0.001
**TC (mg/dL)**	174.07 ± 32.13	186.10 ± 32.57	196.55 ± 33.39	214.20 ± 40.12	<0.001
**TG (mg/dL)**	71.68 (55.76-92.04)	91.15 (72.57-118.59)	117.71 (91.15-153.10)	178.77 (131.87-251.34)	<0.001
**HDL-c (mg/dL)**	67.22 ± 14.76	54.41 ± 9.85	46.56 ± 8.27	37.36 ± 7.69	<0.001
**LDL-c (mg/dL)**	94.52 ± 23.77	113.83 ± 26.07	125.26 ± 29.50	132.30 ± 42.33	<0.001
**non-HDL-c (mg/dL)**	106.84 ± 22.85	131.69 ± 23.55	149.99 ± 25.84	176.84 ± 35.06	<0.001
**non-HDL-c/HDL-c ratio**	1.63 ± 0.32	2.43 ± 0.21	3.24 ± 0.27	4.83 ± 0.95	<0.001
**hsCRP (mg/L)**	0.75 (0.44-1.68)	0.89 (0.49-1.88)	1.07 (0.60-2.18)	1.38 (0.77-2.73)	<0.001
**HbA1c (%)**	5.10 ± 0.60	5.17 ± 0.64	5.26 ± 0.77	5.44 ± 1.06	<0.001
**UA (mg/dL)**	4.26 ± 1.21	4.28 ± 1.19	4.47 ± 1.23	4.79 ± 1.31	<0.001
**HGB (g/dL)**	14.05 ± 2.22	14.21 ± 2.16	14.56 ± 2.27	14.74 ± 2.20	<0.001
**SBP (mmHg)**	127.59 ± 21.04	128.81 ± 21.60	130.81 ± 21.26	134.23 ± 21.62	<0.001
**DBP (mmHg)**	73.66 ± 12.06	74.65 ± 12.09	76.52 ± 11.93	77.98 ± 11.93	<0.001
**BMI (kg/m^2^)**	21.92 ± 3.38	23.05 ± 3.87	24.05 ± 3.80	25.15 ± 3.90	<0.001
**Sex**					<0.001
** Male**	1309 (51.41%)	1136 (44.64%)	1151 (45.21%)	1157 (45.44%)	
** Female**	1237 (48.59%)	1409 (55.36%)	1395 (54.79%)	1389 (54.56%)	
**Hypertension, n (%)**	433 (17.01%)	552 (21.69%)	639 (25.10%)	844 (33.15%)	<0.001
**Diabetes, n (%)**	77 (3.02%)	113 (4.44%)	141 (5.54%)	224 (8.80%)	<0.001
**Malignant tumors, n (%)**	27 (1.06%)	27 (1.06%)	26 (1.02%)	28 (1.10%)	0.995
**CLD, n (%)**	317 (12.45%)	261 (10.26%)	250 (9.82%)	226 (8.88%)	<0.001
**CHD, n (%)**	268 (10.53%)	295 (11.59%)	293 (11.51%)	365 (14.34%)	<0.001
**CKD, n (%)**	190 (7.46%)	179 (7.03%)	147 (5.77%)	161 (6.32%)	0.075
**Mental disease, n (%)**	33 (1.30%)	41 (1.61%)	26 (1.02%)	34 (1.34%)	0.331
**Daily activity, n (%)**	1794 (70.46%)	1681 (66.05%)	1618 (63.55%)	1531 (60.13%)	<0.001
**Smoking status, n (%)**					<0.001
** Never**	1467 (57.62%)	1608 (63.18%)	1595 (62.65%)	1598 (62.77%)	
** Ever**	201 (7.89%)	205 (8.06%)	198 (7.78%)	264 (10.37%)	
** Current**	878 (34.49%)	732 (28.76%)	753 (29.58%)	684 (26.87%)	
**Drinking status, n (%)**					<0.001
** Never**	328 (12.88%)	353 (13.87%)	334 (13.12%)	368 (14.45%)	
** Ever**	1392 (54.67%)	1559 (61.26%)	1642 (64.49%)	1636 (64.26%)	
** Current**	826 (32.44%)	633 (24.87%)	570 (22.39%)	542 (21.29%)	
**Antihypertensive drug use, n (%)**	312 (12.25%)	425 (16.70%)	489 (19.21%)	688 (27.02%)	<0.001
**Antihyperglycemic drug use, n (%)**	53 (2.08%)	66 (2.59%)	98 (3.85%)	158 (6.21%)	<0.001
**Antihyperlipidemic drug use, n (%)**	50 (1.96%)	89 (3.50%)	120 (4.71%)	214 (8.41%)	<0.001

Values are n (%), mean ± SD or medians (quartiles).

BMI, body mass index; WBC, white blood cell; PLT, platelet; hs-CRP, high sensitive C-reactive protein; TG, triglyceride; HGB, hemoglobin; TC, total cholesterol; FPG, fasting plasma glucose; LDL-c, low-density lipoproteins cholesterol; Scr, serum creatinine; HDL-c, high-density lipoprotein cholesterol; non-HDL-c/HDL-c ratio, non-high-density lipoprotein/high-density lipoprotein ratio; non-HDL-c, non-high-density lipoprotein; CKD, Chronic kidney diseases; BUN, blood urea nitrogen; DBP, diastolic blood pressure; SBP, systolic blood pressure; UA, uric acid; CLD, Chronic Lung Diseases; CHD, coronary heart disease; HbA1c, glycosylated hemoglobin.

We also investigated the potential non-linear association between the non-HDL-c/HDL-c ratio and stroke risk by employing the Cox proportional hazards regression model with cubic spline functions and smooth curve fitting. In order to detect any inflection points that suggest a non-linear association, we initially utilized a recursive methodology. Following this, a two-piecewise Cox proportional hazards regression model was developed for both segments of the inflection point. Ultimately, the most optimal model for elucidating the relationship between the non-HDL-c/HDL-c ratio and stroke risk was chosen through a log-likelihood ratio test.

We performed several sensitivity analyses to evaluate the non-linear association between the non-HDL-c/HDL-c ratio and stroke. Considering the strong associations between CKD, diabetes, hypertension, CHD, CLD, and stroke (39–42), we investigated the non-linear relationship between the non-HDL-c/HDL-c ratio and stroke by excluding patients with CKD, diabetes, hypertension, CHD, or CLD from the sensitivity analyses, respectively.

All of the results were made in line with the STROBE statement (43). R statistical software tools (http://www.r-project.org, The R Foundation) and Empower Stats (X&Y Solutions, Inc., Boston, MA, http://www.empowerstats.com) were used for all analyses. The statistical significance was set at P values lower than 0.05(two-sided).

## Results

### Participants’ characteristics

In the final analysis, the participants had an average age of 59.16 ± 9.35 years, with 4735 (46.68%) being male. The initial non-HDL-c/HDL-c ratio had a mean value of 3.03 ± 1.30. During a median follow-up period of 7.0 years, a total of 1191 (11.70%) individuals experienced a stroke event.

The demographic and clinical characteristics of the individuals included in the study are displayed in **Table 1**. We categorized the adults into subgroups based on quartiles of the non-HDL-c/HDL-c ratio (<2.07, ≥2.07 to <2.81, ≥2.81 to <3.75, ≥3.75). When comparing the Q1 group (non-HDL-c/HDL-c ratio <2.07) with the Q4 group (non-HDL-c/HDL-c ratio ≥3.75), we observed significant increases in the values or proportions of WBC, PLT, FPG, Scr, TC, TG, LDL-c, non-HDL-c, hs-CRP, HbA1c, UA, HGB, SBP, DBP, BMI, females, hypertension, diabetes, CHD, ever smokers, ever drinkers, antihypertensive drug use, antihyperglycemic drug use, and antihyperlipidemic drug use. Conversely, opposite outcomes were observed in terms of age, HDL-c, BUN, males, CLD, daily activity, current smokers, and current drinkers for the covariates.

**Figure 2:**
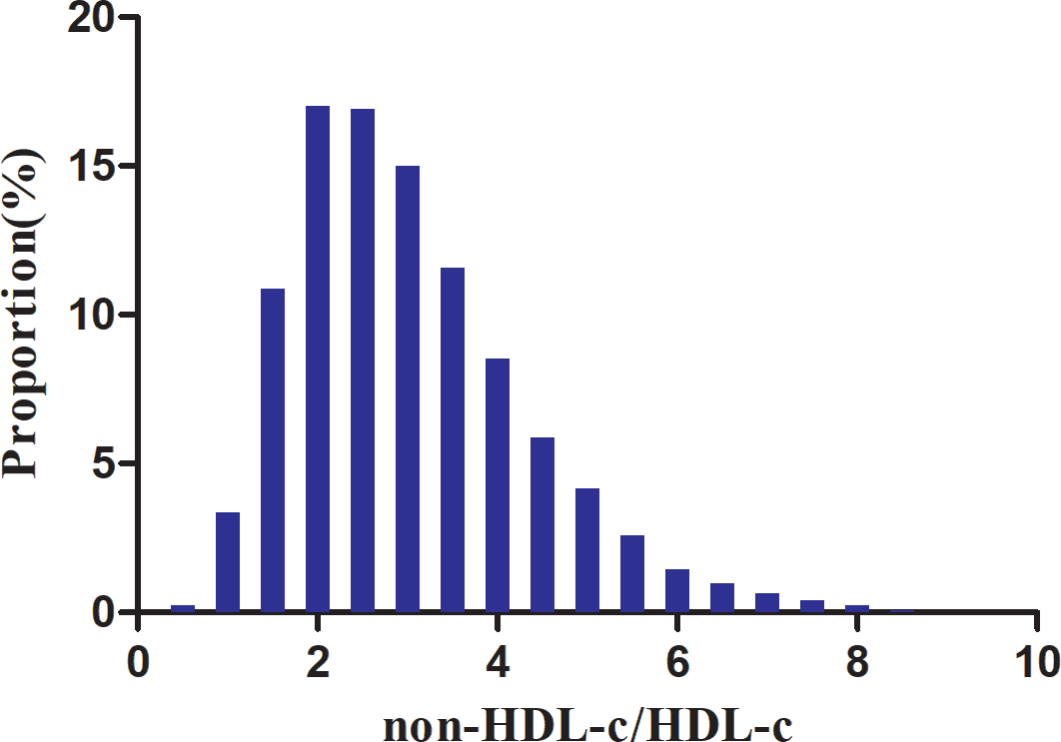
Distribution of non-HDL-c/HDL-c ratio. Depicts the distribution of the normal non-HDL-c/HDL-c ratio, ranging from 0.277 to 8.58, with an average value of 3.03.

According to the data presented in **Figure 2**, the non-HDL-c/HDL-c ratio levels displayed a normal distribution spanning from 0.227 to 8.58, with a mean value of 3.03.

### The incidence rate of stroke

Based on the data presented in **Table 2**, over a median follow-up period of 7.0 years, it was observed that 11.70% (n=1191) of the participants experienced a stroke. The overall cumulative incidence rate for the entire study population was calculated to be 1.92 per 100 person-years. Furthermore, when examining the cumulative incidence rates for each of the four non-HDL-c/HDL-c ratio groups, the rates were found to be 1.44, 1.86, 2.01, and 2.37 per 100 person-years, respectively. The incidence rates for total stroke and each non-HDL-c/HDL-c ratio group were as follows: 11.70% (11.07%-12.32%), 8.88% (7.77%-9.98%), 11.32% (10.08%-12.55%), 12.29% (11.02%-13.57%), and 14.30% (12.94%-15.66%), respectively.

**Table 2:** Incidence rate of stroke.

non-HDL-c/HDL-c ratio	Participants(n)	Stroke events(n)	Incidence rate (95% CI) (%)	Cumulative incidence (Per 100 person-year)
Total	10183	1191	11.70 (11.07-12.32)	1.92
Q1(<2.07)	2546	226	8.88 (7.77-9.98)	1.44
Q2(2.07-2.81)	2546	288	11.32 (10.08-12.55)	1.86
Q3(2.81-3.75)	2546	313	12.29 (11.02-13.57)	2.01
Q4(≥3.75)	2546	364	14.30 (12.94-15.66)	2.37
P for trend			<0.0001	

non-HDL-c/HDL-c ratio, non-high-density lipoprotein/high-density lipoprotein ratio; CI, confidence interval.

### Relationship between the non-HDL-c/HDL-c ratio and the risk of stroke in all participants

**Figure 3:**
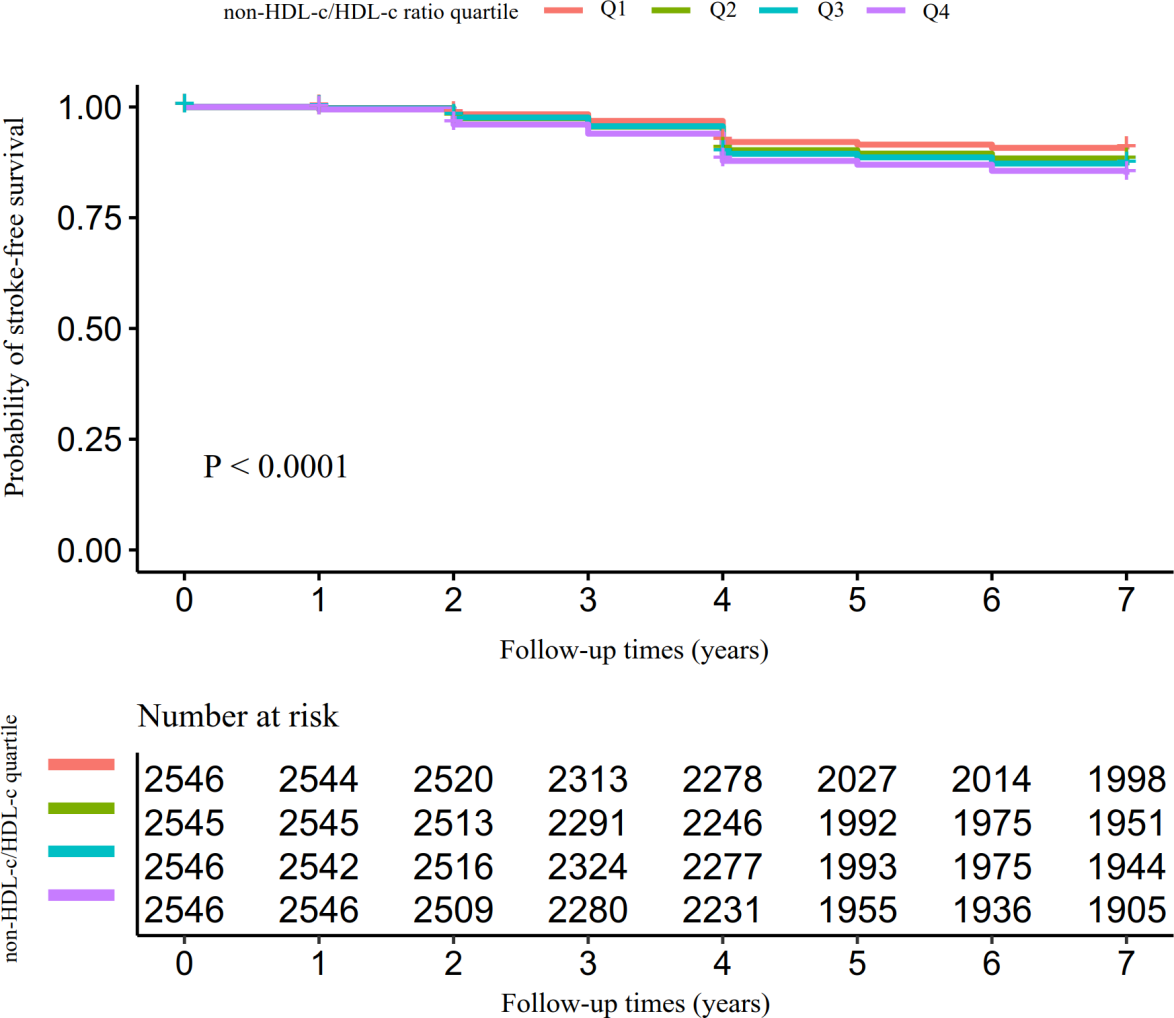
Kaplan–Meier event-free survival curve. Showcases the Kaplan-Meier event-free survival curve. The probability of surviving without stroke significantly varied across the non-HDL-c/HDL-c ratio groups (log-rank test, P<0.0001). As the non-HDL-c/HDL-c ratio increased, the probability of stroke-free survival gradually declined, indicating that the group with the highest non-HDL-c/HDL-c ratio faced the greatest risk of stroke.

**Figure 3** depicts the Kaplan-Meier survival curves, demonstrating the probability of stroke-free survival stratified by groups based on the non-HDL-c/HDL-c ratio. The statistical analysis using the log-rank test revealed a significant difference in stroke-free survival probability among the non-HDL-c/HDL-c ratio groups (P<0.0001). Notably, as the non-HDL-c/HDL-c ratio increased, the probability of stroke-free survival decreased, indicating an elevated risk of stroke in individuals with the highest non-HDL-c/HDL-c ratio.

To examine the relationship between the non-HDL-c/HDL-c ratio and the risk of stroke among all participants, three Cox proportional hazards regression models were constructed (refer to **Table 3**). In the initial model (Model I), a one-unit rise in the non-HDL-c/HDL-c ratio was found to be associated with a 14.1% higher likelihood of experiencing a stroke (HR=1.141, 95% CI 1.096-1.188). Following adjustment for demographic variables in the minimally-adjusted model (Model II), each additional unit of the non-HDL-c/HDL-c ratio was observed to be linked to a 5.7% increase in the risk of stroke (HR=1.057, 95% CI 1.012-1.104). The results obtained from this model demonstrated a statistically significant association between the non-HDL-c/HDL-c ratio and the risk of stroke. However, when considering the fully-adjusted model (Model III) that accounted for all relevant factors, each additional unit of the non-HDL-c/HDL-c ratio was found to be associated with only a 2.2% increase in stroke risk (HR=1.022, 95% CI 0.964-1.083). It is important to note that the confidence intervals indicate that the relationship between the non-HDL-c/HDL-c ratio and stroke risk, as obtained from this model, did not reach statistical significance.

**Table 3:** Relationship between non-HDL-c/HDL-c ratio and the incident stroke in different models.

Exposure	Model I (HR,95%CI, P)	Model II (HR,95%CI, P)	Model III (HR,95%CI, P)
**non-HDL-c/HDL-c ratio**	1.141 (1.096, 1.188) <0.00001	1.057 (1.012, 1.104) 0.01234	1.022 (0.964, 1.083) 0.46623
non-HDL-c/HDL-c ratio quartile			
** Q1**	Ref.	Ref.	Ref.
** Q2**	1.290 (1.083, 1.535) 0.00421	1.204 (1.010, 1.435) 0.03859	1.174 (0.984, 1.401) 0.07478
** Q3**	1.390 (1.171, 1.649) 0.00016	1.255 (1.054, 1.495) 0.01078	1.185 (0.990, 1.419) 0.06464
** Q4**	1.643 (1.391, 1.939) <0.00001	1.245 (1.045, 1.483) 0.01410	1.086 (0.887, 1.331) 0.42449
**P for trend**	<0.00001	0.02015	0.44764

Model I: we did not adjust other covariates.

Model II: we adjust age, gender, BMI, SBP, DBP, smoking and drinking status, daily activity, hypertension, diabetes, CKD, CHD, mental disease, CLD, and malignant tumors.

Model III: we adjust age, gender, BMI, hypertension, diabetes, CKD, CHD, mental disease, CLD, and malignant tumors, daily activity, SBP, DBP, smoking and drinking status, hs-CRP, HGB, TG, Scr, HbA1c, BUN, UA, WBC, PLT, antihypertensive drug use, antihyperglycemic drug use, antihyperlipidemic drug use.

HR, Hazard ratios; CI, confidence; Ref, reference.

Subsequently, we classified the non-HDL-c/HDL-c ratio into quartiles and integrated these discrete variables into our analytical framework. Our findings demonstrated heterogeneity in the effect sizes (HR) among distinct subgroups following the categorization of the non-HDL-c/HDL-c ratio, suggesting the presence of a plausible non-linear relationship between the ratio and the risk of stroke. Furthermore, the inclusion of E-values in our analysis allowed us to evaluate the susceptibility of our findings to potential unmeasured confounding variables. The resulting E-value of 1.19 exceeded the relative risk associated with unmeasured confounders and stroke, indicating that the influence of any unaccounted or unidentified confounding factors on the association between the non-HDL-c/HDL-c ratio and the occurrence of stroke was negligible (35).

Additionally, we present the independent associations between all confounding variables and stroke in **Supplementary Table S2**, where we identified age, sex, antihyperlipidemic drug use, DBP, smoking status, daily activity, CLD, CHD, PLT, and HbA1c as independent influencing factors for stroke.

### The nonlinearity addressed by the generalized additive model

**Figure 4:**
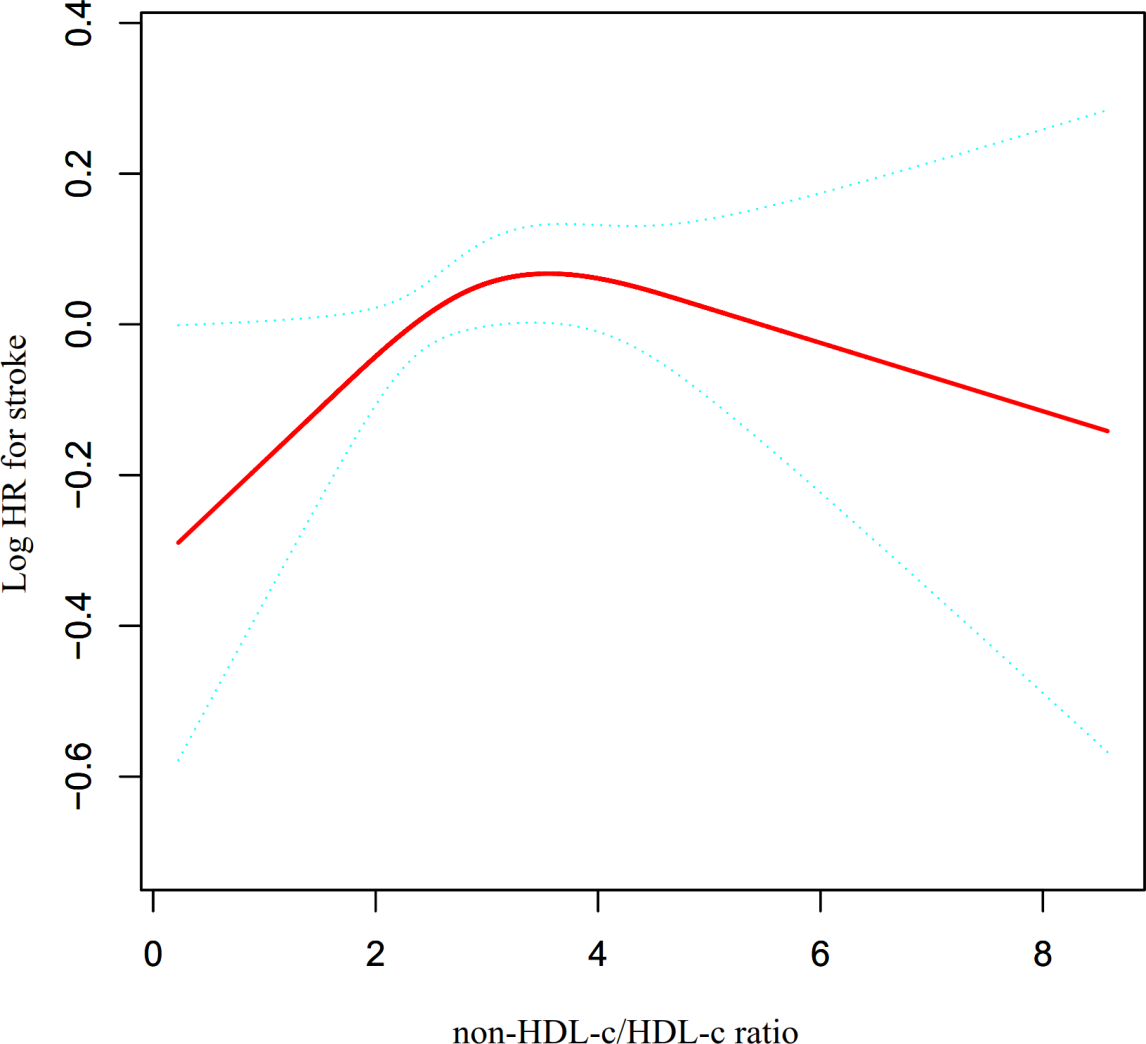
The non-linear relationship between non-HDL-c/HDL-c ratio and the risk of stroke. Presents our utilization of a Cox proportional hazards regression model with cubic spline functions to assess the association between the non-HDL-c/HDL-c ratio and the risk of stroke. The findings indicate a non-linear relationship between the non-HDL-c/HDL-c ratio and stroke, with an inflection point at a non-HDL-c/HDL-c ratio of 2.685.

The utilization of Cox proportional hazards modeling, incorporating cubic spline functions and smooth curve fitting, revealed a non-linear association between the non-HDL-c/HDL-c ratio and the occurrence of stroke (**Figure 4**). This finding was further validated through a log-likelihood ratio test, which yielded a significance level of P < 0.05. To ascertain the inflection point of the non-HDL-c/HDL-c ratio, a recursive technique was employed, resulting in its identification as 2.685. Subsequently, a two-piecewise Cox proportional-hazards regression model was utilized to examine the effect sizes and confidence intervals for the left and right sides of the inflection point. In instances where the non-HDL-c/HDL-c ratio fell below 2.685, for every 1-unit decrease in the non-HDL-c/HDL-c ratio, the likelihood of stroke decreased by 21.4% (HR=1.214, 95% CI: 1.009-1.418). In contrast, when the non-HDL-c/HDL-c ratio exceeded 2.685, there was no statistically significant change in the risk of stroke for each unit decrease in the non-HDL-c/HDL-c ratio (HR: 0.967, 95% CI: 0.897-1.042) (**Table 4**).

**Table 4:** The result of the two-piecewise Cox regression model among all participants.

Incident stroke	HR (95%CI)	P
**Fitting model by standard Cox regression**	1.022 (0.964, 1.083)	0.4662
Fitting model by two-piecewise Cox regression		
** Inflection point of non-HDL-c/HDL-c ratio**	2.685	
** ≤Inflection point**	1.214 (1.039, 1.418)	0.0145
** >Inflection point**	0.967 (0.897, 1.042)	0.3758
**P for log-likelihood ratio test**	0.017	

We adjust age, gender, BMI, hypertension, diabetes, CKD, CHD, mental disease, CLD, and malignant tumors, daily activity, SBP, DBP, smoking and drinking status, CRP, HGB, TG, Scr, HBA1c, BUN, UA, WBC, PLT, antihypertensive drug use, antihyperglycemic drug use, antihyperlipidemic drug use.

HR, Hazard ratios; CI, confidence; Ref, reference.

### Sensitivity analysis

**Figure 5:**
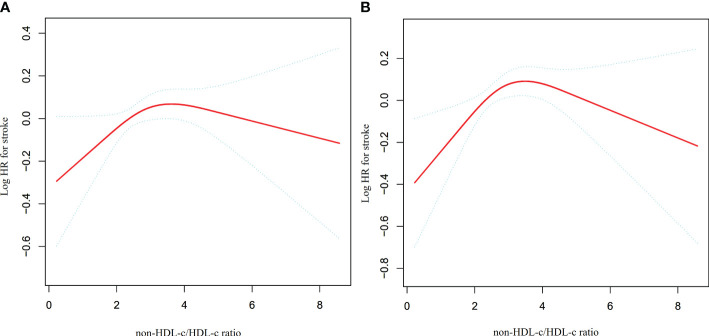
The non-linear relationship between non-HDL-c/HDL-c ratio and stroke in sensitivity analysis. Depicts the findings from our utilization of a Cox proportional hazards regression model with cubic spline functions to examine the association between the non-HDL-c/HDL-c ratio and stroke risk. We excluded participants with CKD (N=7506) or DM (N=9628) in this analysis. The results revealed that a non-linear relationship between the non-HDL-c/HDL-c ratio and stroke persisted among participants without CKD, with an inflection point at a non-HDL-c/HDL-c ratio of 2.752 **(A)**. Furthermore, the results showed that the non-linear relationship between the non-HDL-c/HDL-c ratio and stroke remained significant among participants without diabetes, with an inflection point at a non-HDL-c/HDL-c ratio of 2.746 **(B)**.

A series of sensitivity analyses were conducted to ensure the robustness of the findings. Initially, participants with CKD were excluded from the analysis in order to investigate the non-linear relationship between the non-HDL-c/HDL-c ratio and incident stroke (N=7506). The results confirmed the persistence of the non-linear relationship among participants without CKD (**Figure 5A**). Specifically, the inflection point of the non-HDL-c/HDL-c ratio for participants without CKD was determined to be 2.752. On the left and right sides of the inflection point, the HR and 95% CI were found to be 1.181 (1.011, 1.379) and 0.975 (0.900, 1.056), respectively (**Table 5**).

**Table 5:** The result of the two-piecewise Cox regression model among different participants.

Incident stroke	Model I (HR,95%CI, P)	Model II (HR,95%CI, P)
**Fitting model by standard Cox regression**	1.025 (0.965, 1.090) 0.4198	1.028 (0.966, 1.093) 0.3808
Fitting model by two-piecewise Cox regression		
** Inflection point of non-HDL-c/HDL-c ratio**	2.752	2.746
** ≤Inflection point**	1.181 (1.011, 1.379) 0.0359	1.254 (1.074, 1.464) 0.0043
** >Inflection point**	0.975 (0.900, 1.056) 0.5398	0.955 (0.880, 1.037) 0.2716
**P for log-likelihood ratio test**	0.049	0.005

Model I: Sensitivity analysis in participants without CKD (N=9506); Model II: Sensitivity analysis in participants without diabetes (N=9628).

Model I: We adjusted age, gender, BMI, hypertension, diabetes, CHD, mental disease, CLD, and malignant tumors, daily activity, SBP, DBP, smoking and drinking status, CRP, HGB, TG, Scr, HBA1c, BUN, UA, WBC, PLT, antihypertensive drug use, antihyperglycemic drug use, antihyperlipidemic drug use.

Model II: We adjusted age, gender, BMI, hypertension, CKD, CHD, mental disease, CLD, and malignant tumors, daily activity, SBP, DBP, smoking and drinking status, CRP, HGB, TG, Scr, HBA1c, BUN, UA, WBC, PLT, antihypertensive drug use, antihyperglycemic drug use, antihyperlipidemic drug use.

HR, Hazard ratios; CI, confidence; Ref, reference.

Additionally, a sensitivity analysis was conducted wherein participants with diabetes were excluded. The findings revealed that the non-linear association between the non-HDL-c/HDL-c ratio and stroke remained significant (**Figure 5B**) (N=9628). Specifically, the inflection point of the non-HDL-c/HDL-c ratio was determined to be 2.746. On either side of the inflection point, the hazard ratio and corresponding 95% confidence intervals were calculated as 1.254 (1.074, 1.464) and 0.955 (0.880, 1.037), respectively (**Table 5**).

When we excluded participants with hypertension, CHD, or CLD from the analysis, the observed threshold effect in the relationship between the non-HDL-c/HDL-c ratio and stroke persisted. Specifically, we identified inflection points of 2.677, 2.739, and 2.709 for the non-HDL-c/HDL-c ratio in relation to stroke when considering each condition separately. On the left side of these inflection points, there was a significant and positive association between the non-HDL-c/HDL-c ratio and stroke. However, on the right side of the inflection points, the statistical association between the non-HDL-c/HDL-c ratio and stroke was not significant (see **Supplementary Table S3**).

## Discussion

The incidence of stroke in the general Chinese population has recently increased to 2.47 per 1,000 person-years (44). In our study, the incidence of stroke was observed to be 19.2 per 1,000 person-years, which is higher than previously reported rates. This could be attributed to our focus on investigating the relationship between the non-HDL-c ratio and stroke in middle-aged and older adults (above 45 years) in China. Age is a well-established risk factor for stroke, with those over the age of 65 accounting for 75% of all strokes (45). The stroke incidence rate is expected to be higher in our study population compared to the general population. Moreover, the timing of follow-up visits can also impact the incidence of stroke. The data for our study were obtained from the CHARLS, and due to effective scientific management and follow-up measures, most participants were followed up for 7 years. This prolonged follow-up duration may contribute to the higher incidence of stroke observed in our study. It is essential to acknowledge that this cohort exhibits a significantly higher stroke incidence rate compared to the general population. Therefore, it is crucial to proactively assess potential stroke risk factors in middle-aged and older adults.

Recently, several studies have highlighted the superior predictive abilities of the non-HDL-c and HDL-c combination compared to traditional lipid parameters in relation to atherosclerosis-related diseases (14–16). The non-HDL-c/HDL-c ratio encompasses key information about both atherogenic and antiatherosclerotic lipid particles, making it a potentially better indicator of the balance between these two factors. In the study conducted in Xi’an, China, it was found that elevated non-HDL-C/HDL-C ratios significantly heightened the one-year risk of recurrent stroke in older patients with non-disabling ischemic cerebrovascular events (NICE). Consequently, clinicians should place greater emphasis on this indicator when treating elderly individuals with NICE (46). A separate investigation conducted in Chinese individuals with metabolic syndrome has specifically examined the association between the non-HDL-c/HDL-c ratio and carotid atherosclerosis, revealing a positive correlation. This correlation was found to be particularly significant among women (13). Additionally, a study based on an urban Chinese cohort revealed that the non-HDL-C/HDL-C ratio was notably linked with the stability of carotid plaques, suggesting that it could serve as a valuable marker for the early detection of carotid plaques at elevated risk (47). Given that carotid atherosclerosis plays a significant role in stroke development (48), it is noteworthy that a recent Chinese cohort study discovered a positive association between the non-HDL-c/HDL-c ratio and stroke. After controlling for confounding factors such as age, diabetes, gender, drinking, BMI, smoking, hypertension, and hs-CRP, this connection remained statistically significant (HR=1.24, 95% CI: 1.01-1.52) (17). Our study revealed a positive correlation between the non-HDL-c/HDL-c ratio and the risk of stroke, even after controlling for various confounders such as age, SBP, gender, hypertension, BMI, CKD, DBP, smoking and drinking status, daily activity, diabetes, CHD, mental disease, CLD, and malignant tumors (HR=1.057, 95% CI: 1.012-1.104). However, when considering additional factors, including hs-CRP, HGB, TG, Scr, HbA1c, BUN, UA, WBC, PLT, antihypertensive drug use, antihyperglycemic drug use, and antihyperlipidemic drug use, the association between the non-HDL-c/HDL-c ratio and stroke risk became statistically insignificant (HR=1.022, 95% CI: 0.964-1.083). Several reasons may contribute to the discrepancy between our findings and previous studies. Firstly, the study population differed significantly: their research primarily focused on the general Chinese population with a median age of 51 years, whereas our study targeted middle-aged and older adults with a median age of 58 years. Secondly, their study did not consider the impact of renal function, TG, UA, PLT, and HGB on the relationship between the non-HDL-c ratio and incident stroke when adjusting covariates, unlike our research. However, previous studies have identified these variables as stroke risk factors (18–22). Furthermore, it is imperative to recognize that the application of linear regression analysis may be susceptible to the impact of non-linear associations, resulting in discrepancies in the established linear relationship. In essence, it is plausible that a non-linear correlation between the non-HDL-c/HDL-c ratio and stroke risk exists, which could elucidate the incongruous findings observed across these investigations. This indicates that the relationship between the non-HDL-c/HDL-c ratio and stroke risk may change as the non-HDL-c/HDL-c ratio fluctuates. Furthermore, our study revealed that when incorporating the categorical variable of the non-HDL-c/HDL-c ratio into the Cox proportional risk model, the HRs for Q2, Q3, and Q4 did not demonstrate a sequential increase compared to the reference group (Q1). This further supports the possibility of a non-linear relationship between the non-HDL-c/HDL-c ratio and stroke risk.

Moreover, we would like to highlight that our study is the first to observe a non-linear association between the non-HDL-c/HDL-c ratio and stroke risk among general middle-aged and older adults in China. The reason for considering other variables in participants’ baseline is that they may also impact stroke risk. Comparing individuals with a non-HDL-c/HDL-c ratio<2.685 to those with a ratio≥2.685, it was observed that the latter group generally exhibited higher levels of WBC, PLT, FPG, Scr, TG, LDL-c, hs-CRP, HbA1c, UA, BMI, SBP, and DBP (**Supplementary Table S4**). However, it is important to note that these indicators are closely associated with stroke (18–21, 27, 49–51). When the non-HDL-c/HDL-c ratio exceeds 2.685, the presence of these stroke risk factors diminishes the impact of the ratio on stroke risk. Conversely, when the non-HDL-c/HDL-c ratio is below 2.685, the levels of stroke risk factors such as PLT, FPG, Scr, TG, LDL-c, hs-CRP, HbA1c, UA, BMI, SBP, and DBP tend to be lower, resulting in a weakened effect on stroke. Therefore, at this point, the influence of the non-HDL-c/HDL-c ratio on stroke risk relatively increases. This result is expected to provide a reference for clinicians to control the non-HDL-c/HDL-c ratio. This study provides valuable insights into stroke prevention strategies for individuals with different non-HDL-c/HDL-c ratio statuses. From a therapeutic standpoint, it is advisable to maintain non-HDL-c/HDL-c ratio levels below the inflection point. Reducing the non-HDL-c/HDL-c ratio level can significantly reduce the risk of progression to stroke when the non-HDL-c/HDL-c ratio level is below the inflection point of 2.658. Thus, an abnormal non-HDL-c/HDL-c ratio supports identifying the middle-aged and older Chinese population at high risk of stroke, which would help clinicians plan and initiate the appropriate management strategies in advance. Consequently, this assay holds substantial clinical significance. The findings of this research are anticipated to contribute to future endeavors focused on establishing predictive models for assessing stroke risk.

The interplay of extracellular matrix, inflammatory molecules, endothelial cell dysfunction, and oxidative stress is widely acknowledged as contributing to the development of increased arterial stiffness (52, 53). Hyperlipidemia has been shown to impair vascular endothelial function (54), although the specific effects may vary depending on the components of blood lipids involved. Non-HDL-c encompasses all cholesterol with atherogenic potential in the bloodstream, and elevated levels of non-HDL-c have been linked to damage in vascular endothelial function (54, 55). As a result, reducing non-HDL-C levels is a key objective in the treatment of atherosclerosis (9). HDL-c assumes a protective role in the pathogenesis of atherosclerosis (56) by exerting anti-inflammatory and anti-oxidative effects and safeguarding vascular endothelial cells through the transport of antioxidant enzymes such as paraoxonase-1 (PON1), lecithin cholesterol acyltransferase (LCAT), and platelet-activating factor acetylhydrolase (PAF-AH). Notably, PON1 has been identified as a key mediator of HDL-c’s potential anti-atherogenic properties (57–59). The aforementioned studies highlight the crucial involvement of HDL-c and non-HDL-c in the functional integrity of endothelial cells, substantiating their role in atherosclerosis development. Consequently, the non-HDL-c/HDL-c ratio encapsulates information regarding atherogenic and anti-atherogenic lipid particles.

We highlight several strengths of our study. Firstly, we incorporated both categorical and continuous variables of the non-HDL-c/HDL-c ratio to evaluate its association with stroke risk. This approach minimizes information loss and enables us to quantify the relationship more effectively. Secondly, the implementation of multiple imputations was employed to effectively address missing data, resulting in enhanced statistical power and mitigated potential bias stemming from the lack of covariate information. Thirdly, our study constitutes a noteworthy progression in the examination of nonlinearity, surpassing prior investigations. Specifically, we successfully identified a non-linear correlation between the non-HDL-c/HDL-c ratio and the risk of stroke among middle-aged and older individuals in China. Finally, the robustness of our findings was ensured by conducting a series of sensitivity analyses. These analyses encompassed the calculation of E-values to evaluate the likelihood of unmeasured confounding and the reexamination of the non-linear correlation between the non-HDL-c/HDL-c ratio and the risk of stroke by excluding individuals with CKD, diabetes, hypertension, CHD, or CLD.

It is important to take into account any potential restrictions. Firstly, the study population consisted of middle-aged and elderly Chinese individuals, so further validation is required to generalize these findings to younger populations and other ethnicities. Secondly, it should be noted that the health information provided, encompassing chronic diseases, relied on participant self-reporting. Nevertheless, it is crucial to acknowledge the potential lack of awareness among certain participants regarding their underlying conditions. To address this potential bias, a comprehensive approach was adopted, involving the collection of various measures, including laboratory tests and treatment records. Thirdly, it is important to highlight that our measurements were conducted at a singular time, limiting our ability to evaluate longitudinal trends or alterations in the non-HDL-c/HDL-c ratio over time. Fourthly, as with any observational research, despite adjusting for known potential confounders, uncontrolled or unmeasured confounding factors, such as diet, may still exist. Nevertheless, we estimated E-values to evaluate the potential impact of unmeasured confounders and found them unlikely to significantly affect the results of our study. In the future, we can consider designing our studies or collaborating with other researchers to collect sufficient information on diet and daily activity, thereby minimizing the factors affecting the results’ reliability. Fifthly, it is important to acknowledge that this study is observational and cannot establish a causal relationship between the non-HDL-c/HDL-c ratio and stroke risk. Instead, it demonstrates an association between the two variables. Finally, the generalizability of conclusions drawn solely from data mining may be questionable. To enhance the universality of our results, we plan to collect more cohort data from diverse populations and environments to repeat the analysis and validate our findings. This can reduce data biases and increase the reliability of the results. If circumstances permit, we would very much like to validate our research findings in a clinical setting, which would undoubtedly further demonstrate the clinical significance of our conclusions.

## Conclusion

This study presents findings that reveal a non-linear association between the non-HDL-c/HDL-c ratio and the risk of stroke in the middle-aged and elderly population of China. Notably, a saturation effect is observed, suggesting that the relationship reaches a plateau at a specific threshold of the non-HDL-c/HDL-c ratio. More specifically, when the non-HDL-c/HDL-c ratio falls below 2.685, a significant positive correlation with stroke risk is evident. These findings have important implications for stroke prevention in individuals with varying non-HDL-c/HDL-c ratio levels. Going forward, this study serves as a reference for guiding interventions aimed at reducing stroke incidence. From a therapeutic standpoint, it is advisable to maintain non-HDL-c/HDL-c ratio levels below the inflection point identified in this study. However, the limitations inherent to our study design, including the sample size, observational nature, and follow-up duration, may constrain the confidence with which we can assert our results. Further research is needed to validate and expand upon our results, particularly through studies that can address these limitations with larger sample sizes, longer follow-up periods, and potentially experimental designs that could better establish causality.

## Data availability statement

The datasets presented in this study can be found in online repositories. The names of the repository/repositories and accession number(s) can be found below: The data can be accessed at http://www.isss.pku.edu.cn/cfps/.

## Ethics statement

The studies involving humans were approved by Biomedical Ethics Review Committee at Peking University (IRB00001052–11015). The studies were conducted in accordance with the local legislation and institutional requirements. The participants provided their written informed consent to participate in this study.

## Author contributions

LW: Conceptualization, Data curation, Methodology, Software, Writing – original draft. YH: Formal analysis, Investigation, Software, Writing – original draft. CC: Conceptualization, Methodology, Software, Writing – original draft. HH: Conceptualization, Funding acquisition, Software, Supervision, Validation, Writing – original draft, Writing – review & editing. HL: Conceptualization, Data curation, Formal analysis, Investigation, Methodology, Software, Supervision, Writing – original draft, Writing – review & editing.
