# Supplementary material for: The non-linear link between non-high-density lipoprotein to high-density lipoprotein ratio and the risk of stroke in middle-aged and older adults in Chinese: a prospective cohort study from the China health and retirement longitudinal study
Source: Front Endocrinol (Lausanne). 2024 Jan 15;14:1303336. doi: 10.3389/fendo.2023.1303336 (PMC10823364; doi:10.3389/fendo.2023.1303336)
Supplement: Supplementary file 1 [file Table_1.docx]

**The non-linear link between non-high-density lipoprotein to high-density lipoprotein ratio and the risk of stroke in middle-aged and older adults in Chinese: A retrospective cohort study from the China Health and Retirement Longitudinal Study**

**Running title: non-HDL-c/HDL-c ratio and stroke**

**Lanbo Wang^1#^, Yong Han^2,3#^, Changchun Cao^4#^, Haofei Hu^5,6*^, Han Li^7*^**

^1^Department of Radiology, Shengjing Hospital of China Medical University, Shenyang 110004, Liaoning Province, China

^2^Department of Emergency, Shenzhen Second People’s Hospital, Shenzhen 518000, Guangdong Province, China

^3^Department of Emergency, The First Affiliated Hospital of Shenzhen University, Shenzhen 518000, Guangdong Province, China

^4^Department of Rehabilitation, Shenzhen Dapeng New District Nan’ao People’s Hospital, Shenzhen 518000, Guangdong Province, China

^5^Department of Nephrology, Shenzhen Second People’s Hospital, Shenzhen 518000, Guangdong Province, China

^6^Department of Nephrology, The First Affiliated Hospital of Shenzhen University, Shenzhen 518000, Guangdong Province, China

^7^Department of Neurosurgery, Shengjing Hospital of China Medical University, Shenyang 110004, Liaoning Province, China

**# Lanbo Wang, Yong Han and Changchun Cao have contributed equally to this work.**

***Corresponding author**

**Haofei Hu**

Department of Nephrology,

Shenzhen Second People’s Hospital,

No.3002 Sungang Road, Futian District,

Shenzhen 518000,

Guangdong Province,

China

E-mail: [huhaofei0319@126.com](mailto:huhaofei0319@126.com)

***Corresponding author**

**Han Li**

Department of Neurosurgery,

Shengjing Hospital of China Medical University,

No.36 Sanhao Street,

Shenyang 110004,

Liaoning Province,

China

E-mail: leoincmu@outlook.com

**Table S1. Collinearity diagnostics steps.**

| Variable | VIF  Step 1 |
| --- | --- |
|  |  |
| Gender | 2.2 |
| Age(years) | 1.4 |
| CKD | 1.0 |
| CHD | 1.1 |
| diabetes | 2.9 |
| WBC (10^9/L) | 1.2 |
| Malignant tumors | 1.0 |
| CLD | 1.0 |
| Mental disease | 1.0 |
| Daily activity | 1.1 |
| Smoking status | 1.8 |
| Drinking status | 1.1 |
| hypertension | 3.4 |
| PLT (10^9/L) | 1.1 |
| BUN (mg/dL) | 1.2 |
| UA (mg/dL) | 1.5 |
| HGB (g/dL) | 1.2 |
| HbA1c (%) | 1.2 |
| hs-CRP (mg/L) | 1.1 |
| TG (mg/dL) | 1.1 |
| Scr (mg/dL) | 1.5 |
| BMI (kg/m^2^) | 1.3 |
| SBP (mmHg) | 2.8 |
| DBP (mmHg) | 2.6 |
| Antihypertensive drug use | 3.3 |
| Antihyperglycemic drug use | 3.0 |
| Antihyperlipidemic drug use | 1.1 |

BMI, body mass index; WBC, white blood cell; PLT, platelet; hs-CRP, high-sensitive C-reactive protein; TG, triglyceride; HGB, hemoglobin; Scr, serum creatinine; CKD, Chronic kidney diseases; BUN, blood urea nitrogen; DBP, diastolic blood pressure; SBP, systolic blood pressure; UA, uric acid; CLD, Chronic Lung Diseases; CHD, coronary heart disease; HbA1c, glycosylated hemoglobin.

Abbreviation: VIF: variance inflation factor; VIF = 1/(1-R^2^).

Note: The variables with VIF>5 will be regarded as collinear variables and cannot be included in the multiple regression model.

**Table S2. Factors influencing stroke outcomes**

| Exposure | Univariable (HR,95%CI, P) | Multivariable (HR,95%CI, P) |
| --- | --- | --- |
| non-HDL-c/HDL-c ratio | 1.141 (1.096, 1.188) <0.00001 | 1.022 (0.964, 1.083) 0.46623 |
| Age(years) | 1.031 (1.025, 1.037) <0.00001 | 1.019 (1.012, 1.027) <0.00001 |
| Sex |  |  |
| Male | 1.0 | 1.0 |
| Female | 1.132 (1.009, 1.269) 0.03446 | 1.206 (1.009, 1.442) 0.03989 |
| BMI (kg/m^2^) | 1.048 (1.035, 1.060) <0.00001 | 1.013 (0.998, 1.029) 0.09580 |
| WBC (10^9/L) | 1.038 (1.014, 1.063) 0.00186 | 1.008 (0.977, 1.040) 0.63610 |
| Antihyperlipidemic drug use |  |  |
| No | 1.0 | 1.0 |
| Yes | 2.821 (2.355, 3.379) <0.00001 | 1.221 (1.001, 1.488) 0.04895 |
| Antihypertensive drug use |  |  |
| No | 1.0 | 1.0 |
| Yes | 2.826 (2.513, 3.179) <0.00001 | 1.215 (0.967, 1.526) 0.09514 |
| Antihyperglycemic drug use |  |  |
| No | 1.0 | 1.0 |
| Yes | 2.398 (1.934, 2.973) <0.00001 | 0.972 (0.657, 1.438) 0.88590 |
| SBP (mmHg) | 1.014 (1.011, 1.016) <0.00001 | 1.002 (0.998, 1.006) 0.45075 |
| DBP (mmHg) | 1.018 (1.013, 1.023) <0.00001 | 1.008 (1.001, 1.015) 0.02856 |
| Smoking status |  |  |
| Never | 1.0 | 1.0 |
| Ever | 1.452 (1.208, 1.746) 0.00007 | 1.241 (1.005, 1.533) 0.04484 |
| Current | 0.951 (0.835, 1.083) 0.44637 | 1.197 (1.013, 1.416) 0.03499 |
| Drinking status |  |  |
| Never | 1.0 | 1.0 |
| Ever | 0.833 (0.711, 0.975) 0.02316 | 0.881 (0.742, 1.046) 0.14749 |
| Current | 0.688 (0.571, 0.829) 0.00009 | 0.882 (0.728, 1.069) 0.20088 |
| Daily activity |  |  |
| No | 1.0 | 1.0 |
| Yes | 0.641 (0.572, 0.719) <0.00001 | 0.857 (0.760, 0.967) 0.01203 |
| Hypertension |  |  |
| No | 1.0 | 1.0 |
| Yes | 2.691 (2.400, 3.017) <0.00001 | 1.249 (0.997, 1.565) 0.05323 |
| Diabetes |  |  |
| No | 1.0 | 1.0 |
| Yes | 2.297 (1.911, 2.761) <0.00001 | 1.212 (0.871, 1.688) 0.25395 |
| Malignant tumors |  |  |
| No | 1.0 | 1.0 |
| Yes | 1.380 (0.842, 2.260) 0.20087 | 1.079 (0.656, 1.774) 0.76463 |
| CLD |  |  |
| No | 1.0 | 1.0 |
| Yes | 1.698 (1.450, 1.988) <0.00001 | 1.204 (1.023, 1.418) 0.02586 |
| CHD |  |  |
| No | 1.0 | 1.0 |
| Yes | 5.534 (4.925, 6.219) <0.00001 | 3.990 (3.505, 4.543) <0.00001 |
| CKD |  |  |
| No | 1.0 | 1.0 |
| Yes | 1.658 (1.374, 2.001) <0.00001 | 1.148 (0.945, 1.395) 0.16479 |
| Mental disease |  |  |
| No | 1.0 | 1.0 |
| Yes | 1.172 (0.736, 1.867) 0.50408 | 0.872 (0.545, 1.394) 0.56657 |
| PLT (10^9/L) | 1.001 (1.000, 1.002) 0.01195 | 1.001 (1.000, 1.002) 0.04624 |
| BUN (mg/dL) | 1.005 (0.992, 1.018) 0.45099 | 0.999 (0.986, 1.013) 0.94066 |
| Scr (mg/dL) | 1.364 (1.159, 1.605) 0.00019 | 1.053 (0.759, 1.461) 0.75610 |
| TG(mg/dL) | 1.002 (1.001, 1.002) <0.00001 | 1.000 (0.999, 1.001) 0.54221 |
| hsCRP(mg/L) | 1.010 (1.005, 1.016) 0.00021 | 1.006 (1.000, 1.013) 0.05750 |
| HbA1c (%) | 1.197 (1.134, 1.262) <0.00001 | 1.074 (1.006, 1.147) 0.03244 |
| UA (mg/dL) | 1.079 (1.032, 1.128) 0.00088 | 1.017 (0.963, 1.073) 0.54687 |
| HGB (g/dL) | 1.023 (0.998, 1.049) 0.07284 | 1.010 (0.981, 1.039) 0.49906 |

BMI, body mass index; WBC, white blood cell; PLT, platelet; hs-CRP, high sensitive C-reactive protein; TG, triglyceride; HGB, hemoglobin; Scr, serum creatinine; non-HDL-c/HDL-c ratio, non-high-density lipoprotein/high-density lipoprotein ratio; CKD, Chronic kidney diseases; BUN, blood urea nitrogen; DBP, diastolic blood pressure; SBP, systolic blood pressure; UA, uric acid; CLD, Chronic Lung Diseases; CHD, coronary heart disease; HbA1c, glycosylated hemoglobin.

HR, Hazard ratios; CI: confidence interval, Ref: reference.

**Table S3. The result of the two-piecewise Cox regression model among other different participants**

| Incident stroke | Model III (HR,95%CI, P) | Model IV (HR,95%CI, P) | Model V (HR,95%CI, P) |
| --- | --- | --- | --- |
| Fitting model by standard Cox regression | 1.045 (0.966, 1.130) 0.2753 | 1.062 (0.986, 1.143) 0.1128 | 1.011 (0.949, 1.077) 0.7326 |
| Fitting model by two-piecewise Cox regression |  |  |  |
| Inflection point of non-HDL-c/HDL-c ratio | 2.677 | 2.739 | 2.709 |
| ≤Inflection point | 1.222 (1.000, 1.493) 0.0495 | 1.240 (1.022, 1.505) 0.0294 | 1.229 (1.036, 1.458) 0.0181 |
| >Inflection point | 0.987 (0.889, 1.095) 0.8021 | 1.008 (0.916, 1.109) 0.8745 | 0.951 (0.877, 1.031) 0.2229 |

Model III: Sensitivity analysis in participants without hypertension (N=7715); Model IV: Sensitivity analysis in participants without CHD (N=8962); Model V: Sensitivity analysis in participants without CLD (N=9129)

Model III: We adjusted age, gender, BMI, CKD, diabetes, CHD, mental disease, CLD, and malignant tumors, daily activity, SBP, DBP, smoking and drinking status, CRP, HGB, TG, Scr, HBA1c, BUN, UA, WBC, PLT, antihypertensive drug use, antihyperglycemic drug use, antihyperlipidemic drug use

Model IV: We adjusted age, gender, BMI, hypertension, CKD, diabetes, mental disease, CLD, and malignant tumors, daily activity, SBP, DBP, smoking and drinking status, CRP, HGB, TG, Scr, HBA1c, BUN, UA, WBC, PLT, antihypertensive drug use, antihyperglycemic drug use, antihyperlipidemic drug use

Model V: We adjusted age, gender, BMI, hypertension, CKD, diabetes, mental disease, CHD, and malignant tumors, daily activity, SBP, DBP, smoking and drinking status, CRP, HGB, TG, Scr, HBA1c, BUN, UA, WBC, PLT, antihypertensive drug use, antihyperglycemic drug use, antihyperlipidemic drug use

HR, Hazard ratios; CI: confidence, Ref: reference;

**Table S4. The characteristics of participants on both sides of the inflection point.**

| Non-HDL-c/HDL-c ratio | <2.685 | >=2.685 | P-value |
| --- | --- | --- | --- |
| Participants | 4718 | 5465 |  |
| Age (years) | 59.55 ± 9.69 | 58.83 ± 9.03 | <0.001 |
| WBC (10^9/L) | 6.00 ± 1.88 | 6.44 ± 2.35 | <0.001 |
| PLT (10^9/L) | 204.28 ± 73.22 | 217.03 ± 71.95 | <0.001 |
| BUN (mg/dL) | 16.08 ± 4.96 | 15.48 ± 4.29 | <0.001 |
| FPG (mg/dL) | 104.12 ± 26.42 | 114.15 ± 40.30 | <0.001 |
| Scr (mg/dL) | 0.77 ± 0.28 | 0.79 ± 0.19 | <0.001 |
| TC (mg/dL) | 179.43 ± 32.77 | 204.21 ± 37.90 | <0.001 |
| TG (mg/dL) | 87.89 ± 40.19 | 164.61 ± 97.49 | <0.001 |
| HDL-c (mg/dL) | 61.65 ± 14.08 | 42.53 ± 9.44 | <0.001 |
| LDL-c (mg/dL) | 103.09 ± 26.47 | 128.03 ± 36.15 | <0.001 |
| Non-HDL-c (mg/dL) | 117.79 ± 25.88 | 161.68 ± 33.67 | <0.001 |
| Non-HDL-c/HDL-c ratio | 1.97 ± 0.46 | 3.94 ± 1.07 | <0.001 |
| Hs-CRP (mg/L) | 2.53 ± 7.46 | 2.82 ± 6.96 | 0.043 |
| HbA1c (%) | 5.13 ± 0.63 | 5.34 ± 0.91 | <0.001 |
| UA (mg/dL) | 4.27 ± 1.20 | 4.60 ± 1.28 | <0.001 |
| HGB (g/dL) | 14.11 ± 2.19 | 14.62 ± 2.24 | <0.001 |
| SBP (mmHg) | 128.14 ± 21.37 | 132.28 ± 21.48 | <0.001 |
| DBP (mmHg) | 74.10 ± 12.15 | 77.09 ± 11.91 | <0.001 |
| BMI (kg/m^2^) | 22.41 ± 3.67 | 24.52 ± 3.89 | <0.001 |
| Sex |  |  | 0.001 |
| Male | 2283 (48.39%) | 2470 (45.20%) |  |
| Female | 2435 (51.61%) | 2995 (54.80%) |  |
| Hypertension, n (%) | 901 (19.10%) | 1567 (28.67%) | <0.001 |
| Diabetes, n (%) | 175 (3.71%) | 380 (6.95%) | <0.001 |
| Malignant tumors, n (%) | 48 (1.02%) | 60 (1.10%) | 0.692 |
| CLD, n (%) | 541 (11.47%) | 513 (9.39%) | <0.001 |
| CHD, n (%) | 522 (11.06%) | 699 (12.79%) | 0.007 |
| CKD, n (%) | 345 (7.31%) | 332 (6.08%) | 0.012 |
| Mental disease, n (%) | 69 (1.46%) | 65 (1.19%) | 0.228 |
| Daily activity, n (%) | 3240 (68.67%) | 3384 (61.92%) | <0.001 |
| Smoking status, n (%) |  |  | <0.001 |
| Never | 2847 (60.34%) | 3421 (62.60%) |  |
| Ever | 375 (7.95%) | 493 (9.02%) |  |
| Current | 1496 (31.71%) | 1551 (28.38%) |  |
| Drinking status, n (%) |  |  | <0.001 |
| Never | 626 (13.27%) | 757 (13.85%) |  |
| Ever | 2720 (57.65%) | 3509 (64.21%) |  |
| Current | 1372 (29.08%) | 1199 (21.94%) |  |
| Antihypertensive drug use, n (%) | 673 (14.26%) | 1241 (22.71%) | <0.001 |
| Antihyperglycemic drug use, n (%) | 113 (2.40%) | 262 (4.79%) | <0.001 |
| Antihyperlipidemic drug use, n (%) | 127 (2.69%) | 346 (6.33%) | <0.001 |

Values are n (%), mean±SD or medians (quartiles)

BMI, body mass index; WBC, white blood cell; PLT, platelet; hs-CRP, high sensitive C-reactive protein; TG, triglyceride; HGB, hemoglobin; TC, total cholesterol; FPG, fasting plasma glucose; LDL-c, low-density lipoproteins cholesterol; Scr, serum creatinine; HDL-c, high-density lipoprotein cholesterol; non-HDL-c/HDL-c ratio, non-high-density lipoprotein/high-density lipoprotein ratio; non-HDL-c, non-high-density lipoprotein; CKD, Chronic kidney diseases; BUN, blood urea nitrogen; DBP, diastolic blood pressure; SBP, systolic blood pressure; UA, uric acid; CLD, Chronic Lung Diseases; CHD, coronary heart disease; HbA1c, glycosylated hemoglobin.
